# Inhibitory activity of 9-phenylcyclohepta[*d*]pyrimidinedione derivatives against different strains of HIV-1 as non-nucleoside reverse transcriptase inhibitors

**DOI:** 10.1186/1743-422X-8-230

**Published:** 2011-05-15

**Authors:** Yang Huang, Xiaowei Wang, Xiaoling Yu, Lin Yuan, Ying Guo, Weisi Xu, Tiejun Liu, Junyi Liu, Yiming Shao, Liying Ma

**Affiliations:** 1 State Key Laboratory for Infection Disease Prevention and Control, National Center for AIDS/STD Control and Prevention (NCAIDS), Chinese Center for Disease Control and Prevention (CDC), China; 2 Department of Chemical Biology, School of Pharmaceutical Science, State Key Laboratory of Natural and Biomimetic Drugs, Peking University, China

## Abstract

**Background:**

The non-nucleoside reverse transcriptase inhibitor (NNRTI), as a major component of the highly active antiretroviral therapy (HAART) to HIV-1 (human immunodeficiency virus type 1) infected patients, required the development of new NNRTIs with improved resistance profile and decreased toxicity. Therefore, a series of novel compounds, 9-phenylcyclohepta[d]pyrimidinedione derivatives (PCPs), were designed based on the chemical structure of TNK-651, to detect anti-HIV-1 activity.

**Results:**

1-[(benzyloxy)methyl]-9-phenyl-cyclohepta[d] pyrimidinedione (BmPCP) among four PCPs has antiviral activity on laboratory-adapted HIV strains (HIV-1 SF33). The results showed 50% inhibition concentrations (IC_50_s) of BmPCP were 0.34 μM, 1.72 μM and 1.96 μM on TZM-bl, peripheral blood mononuclear cells (PBMCs) and MT4, respectively. It was also effective against infection by the predominant HIV-1 isolates in China, with IC_50_s at low μM levels. Its selectivity index (SI) ranged from 67 to 266 in different cells. The results of time-of-addition assay demonstrated that BmPCP inhibited HIV-1 infection by targeting the post entry of the HIV-1 replication cycle. For inhibition of HIV-1 reverse transcriptase activity, the IC_50 _values of BmPCP and NVP were 1.51 and 3.67 μM, respectively.

**Conclusions:**

BmPCP with a novel structure acts as a NNRTI to inhibit HIV-1 replication and can serve as a lead compound for further development of new anti-HIV-1 drugs.

## Background

Human immunodeficiency virus type 1 (HIV-1) reverse transcriptase (RT) converts single-stranded viral RNA into a double-stranded proviral DNA. Reverse transcription is a necessary step in the HIV-1 replication cycle[1]. Therefore, the inhibition of reverse transcriptase (RT) has been one of important targets in inhibiting the replication of HIV-1 and RT inhibitors have been the primary therapeutic strategies in AIDS patient treatment[2,3]. So far, two classes of RT inhibitors are available for the treatment of HIV-1 infection: 1) nucleoside RT inhibitors (NRTIs), such as zidovudine (AZT) and lamivudine (3TC), which bind directly to the active site of RT polymerase and terminate DNA synthesis after incorporation into the newly synthesized DNA, and 2) non-nucleoside RT inhibitors (NNRTIs) that bind to the hydrophobic pocket within the polymerase domain of the p66 RT subunit, resulting in inhibition of RT activity[4].

There are nevirapine (NVP), delavirdine (DLV), and efavirenz (EFV) to be approved by American Food and Drug Administration (FDA) for clinical application. NNRTIs, a major component of the highly active antiretroviral therapy (HAART) are included [5]. Application of such NNRTIs in combination with nucleoside analogues is highly effective in inhibiting HIV-1 replication. However, drug resistance and side effort can cause antiviral therapeutic failure. In China it was reported that the rates of resistance to NRTIs and NNRTIs were 1.6% and 2.1%, respectively [6]. Also there was a high level of cross-drug resistance to HIV-1 RTIs (reverse transcriptase inhibitors) among Chinese AIDS (Acquired Immune Deficiency Syndrome) patients harboring resistant strains [7,8]. Therefore, it is essential to develop new NNRTIs with improved drug resistance and decreased toxicity.

To develop new NNRTIs, a series of 9-phenylcyclohepta[d]pyrimidinedione derivatives were designed and synthesized in the School of Pharmaceutical Sciences, Peking University based on TNK-651, a potent NNRTI. According to structure-activity relationships (SARs), studies of the crystal structure of the RT complex with TNK-651 inhibitor suggest that cycloheptyl group would adjust the aromatic ring with an effective conformation to the plane of the pyrimidine ring, which could improve the antiviral activity. 1-[(benzyloxy)methyl]-9-phenyl-cyclohepta[d]pyrimidinedione (BmPCP) is from 9-phenyl-cyclohepta[d]pyrimidinedione derivatives [9]. This study aims to evaluate BmPCP anti-HIV-1 activity and explore its putative mechanism of action.

## Results

### Identification of BmPCP as a RT inhibitor from 9-phenylcyclohepta[d] pyrimidinedione derivatives

**Figure 1:**
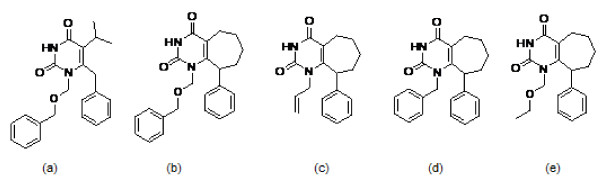
**The structural formulas of TNK651 and PCP derivatives**. (a)TNK-651, (b)1-[(benzyloxy) methyl]-9-phenyl-cyclohepta[d]pyrimidinedione (BmPCP), (c) 1-Allyl -9-phenyl-cyclohepta[d ]pyrimidinedione(APCP), (d)1-Benzyl -9-phenyl-cyclohepta[d]pyrimidinedione(BPCP), (e)1-(Ethoxymethyl)- 9-phenyl-cyclohepta[d]pyrimidinedione (EPCP)

To identify new NNRTIs, a series of cyclohepta[d]pyrimidine derivatives by using TNK-651 (Figure 1a) as a template were designed and synthesized by the School of Pharmaceutical Sciences, Peking University. The compounds were 1-[(benzyloxy)methyl]-9-phenyl-cyclohepta[d]pyrimidinedione(BmPCP), 1-Allyl-9-phenyl-cyclohepta[d]pyrimidinedione(APCP), 1-Benzyl-9-phenyl -cyclohepta[d]pyrimidinedione (BPCP), 1-(Ethoxymethyl)- 9-phenyl-cyclohepta[d] pyrimidinedione (EPCP) and their molecular weights were 376.45, 296.36, 346.42 and 314.38 separately.(Figure 1b,c,d,e)

PCPs were tested for their inhibition on a commonly used laboratory-adapted reference strain-HIV-1SF33 in different cells. We found that BmPCP (molecular weight of 376.45), which has a characteristic seven-alicyclic benzene ring conformation (Figure 1b), exhibited the highest inhibitory potency and lowest toxicity among all four compounds. 50% cytoxicity concentrations (CC_50_s) of BmPCP are 90.26 μM and 129.29 μM respectively while 50% inhibition concentrations (IC50s) are 1.96 μM and 0.34 μM on MT4 and TZM-bl cells. Selectivity index (SI = CC_50_/IC_50_) is 84 and 266 on above both cells. (Table 1) Therefore, BmPCP was selected for further testing antiviral activity to different clinical HIV isolates and target in this study.

**Table 1:** The inhibitory activity of PCPs on infection by HIV-1 SF33

Compounds	Virus/Cell	CC_50_(μM)	IC_50_(μM)	SI^a^
BmPCP	SF33/MT4	165.29	1.96	84
	SF33/TZM-bl	90.26	0.34	266
APCP	SF33/MT4	62	28.6	2.17
	SF33/TZM-bl	50	1.3	38.46
BPCP	SF33/MT4	132.25	114,7	1.15
	SF33/TZM-bl	180	9	20
EPCP	SF33/MT4	29.28	7.5	3.9
	SF33/TZM-bl	22	1.6	13.75

^a^Selectivity index(SI) = CC_50_/IC_50_

### BmPCP is effective in inhibiting viral infection on different cells

We tested the antiviral activity of BmPCP in three cells including peripheral blood mononuclear cells (PBMCs), TZM-b1 and MT4 cells by using HIV-1SF33 and HIV-1 1617-1, a variant bearing K70G, 184V, 75I, 77L, I16Y and I51M mutations, which are resistant to NRTIs (e.g., d4T, DDI and AZT). NVP, a NNRTI was used as a control. The result showed that BmPCP, like NVP, can effectively inhibited HIV-1 infection including HIV-1SF33 and HIV-1 1617-1 strains on PBMCs, TZM-b1 and MT4 cells (Table 2), but it was ineffective in inhibiting infection by HIV-1_IIIB _A17 variant which harbors K103N and Y181C mutations and is highly resistant to NNRTIs.

**Table 2:** The inhibitory activity of BmPCP on infection in different cells

		TZM-bl		PBMCs		MT4	
		
Compounds	HIV-1 strain	IC_50_^a ^(μM)	SI^b^	IC_50 _^a^(μM)	SI^b^	IC_50 _^a^(μM)	SI^b^
BmPCP	1617-1	1.45 ± 0.21	62	1.76 ± 0.23	65	1.47 ± 0.43	128
	SF33	0.34 ± 0.09	266	1.72 ± 0.37	67	1.96 ± 0.58	84
NVP	1617-1	0.16 ± 0.10	-	0.57 ± 0.08	-	0.44 ± 0.12	-
	SF33	0.19 ± 0.02	-	0.69 ± 0.07	-	0.65 ± 0.23	-

^a ^Compounds were tested in triplicate and the data are presented as means ± SD.

^b ^Selectivity index(SI) = CC_50_/IC_50_

### BmPCP exhibits potential inhibitory activity against infection by primary HIV-1 isolates circulating in China

HIV-1CRF07_BC and B' (Thailand B, a kind variant of subtype B) are two epidemic strains in China. Furthermore, to verify the antiviral activity on China clinical isolates, CRF07_BC and B' were chosen. We found that BmPCP was also effective in inhibiting infection of PBMCs by the primary HIV-1 isolates that are circulating in China, although its potency is lower than NVP (Table 3).

**Table 3:** The IC_50 _values of BmPCP against infection by primary HIV-1 isolates circulating in China

			BmPCP		NVP	
			
HIV-1	subtype	Co-receptor	IC_50 _^a^(μM)	SI^b^	IC_50 _^a^(μM)	SI^b^
AH104	B'	X4/R5	0.59 ± 0.34	153	0.007 ± 0.003	29,143
AH259	B'	R5	0.32 ± 0.15	282	0.007 ± 0.001	29,143
AH968	B'	R5	0.44 ± 0.17	205	0.058 ± 0.060	3,517
XJ257	B'/C	R5	0.53 ± 0.07	170	0.211 ± 0.120	967
XJ0793	B'/C	R5	0.25 ± 0.03	361	0.079 ± 0.097	2,582
XJ6291	B'/C	R5	0.57 ± 0.02	158	0.186 ± 0.224	1,097

^a ^Compounds were tested in triplicate and the data are presented as means ± SD.

^b ^Selectivity index(SI) = CC_50_/IC_50_

### BmPCP has low cytotoxicity on different cells used for testing antiviral activity

XTT assay [10] was used to test the cytotoxicity of BmPCP on MT4 cells, human PBMCs (non-stimulated) and TZM-bl cells. BmPCP cytotoxicity on these cells was demonstrated as 50% or 90% cytotoxic concentration (CC_50 _or CC_90_). CC_50 _values are ranging from 90 to 165 μM (Table 4).

**Table 4:** The CC_50 _and CC_90 _values of BmPCP in different cells ^a^

Cells	CC_50_^b ^(μM)	CC_90 _(μM) ^b^
TZM-bl	90.26 ± 2.73	207.93 ± 8.69
PBMC	114.5 ± 9.38	228.56 ± 6.40
MT4	165.29 ± 30.40	382.59 ± 53.52

^a^The compound was tested in triplicate and the data are presented as means ± SD.

^b^XTT assay was used to determine the 50% or 90% cytotoxic concentration (CC_50 _or CC_90_)

### BmPCP targets the post entry of HIV-1 replication cycle

**Figure 2:**
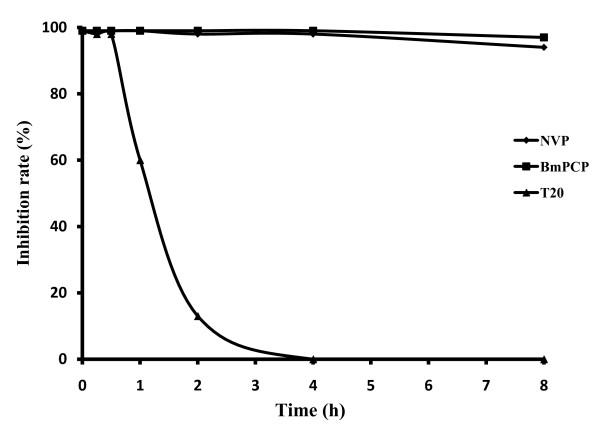
**Time-of-addition assay of compounds**. TZM-bl cells were infected with HIV-1SF33 at 200 TCID_50_. BmPCP(■), NVP(◆) and T-20(▲), were respectively added 0, 0.25, 0.5, 1, 2, 4, and 8 hrs post-infection. Results are the mean inhibition rate in duplicate experiments

To determine the target of BmPCP during HIV-1 replication, we performed a time-of-addition assay by using Enfuvirtide (T20), the HIV-1 fusion inhibitor interfering with entry of the HIV-1 virus into cells, and NVP, a NNRTI, as controls. As shown in Figure 2, unlike T20 which exhibited no inhibitory activity when it was added to the virus culture one hour after viral infection, both BmPCP and NVP demonstrated antiviral activity when they were added to the virus culture even eight hours post-infection. These results suggest that BmPCP is similar to NVP which inhibits HIV-1 infection by targeting the post entry of the HIV-1 replication cycle.

### BmPCP can effectively inhibit HIV-1 reverse transcriptase activity in vitro

**Figure 3:**
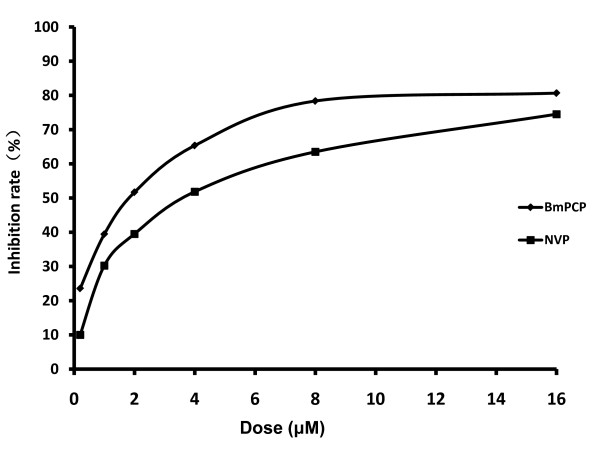
**The inhibition effect of BmPCP and NVP on HIV-RT in vitro**. The inhibition rate of BmPCP and NVP are tested at the dose of 0.2, 1, 2, 4, 8, 16 μM and the results are the mean inhibition rate in duplicate experiments

To further identify the target site of BmPCP during the HIV life cycle, the method based on ELISA RT assay was used to detect the inhibition activity of reverse transcriptase. Oligo(dT) 15 was immobilized via its 5'-terminal phosphate to Covalink-NH microtiter plates. The biotin-dUTP was incorporated by reverse transcriptase. The products were detected and quantified using a colorimetric strep- tavidin-alkaline phosphatase reporter system. The IC_50 _values of BmPCP and NVP were 1.51 and 3.67 μM, respectively, indicating BmPCP directly targets the process of HIV reverse transcriptase. (Figure 3)

## Discussion

The novel cyclohepta[d]pyrimidine derivatives targeting the non-nucleoside reverse transcriptase inhibitor (NNRTI) binding site have been rationally designed based on the structure of the NNRTI binding pocket. Although extensive SARs for non-cyclic analogues (such as MKC-442 and TNK-651) have been obtained, little or no information was available about the annelated series. The key point for the development of such compounds is the right size of the annelated rings, which should not only possess conformational flexibility, but also restrict effectively the rotation and position of the aromatic ring to the plane of the pyrimidine ring. According to structure-activity relationships (SARs), studies of the crystal structure of the RT complex with TNK-651 inhibitor suggest that cycloheptyl group would adjust the aromatic ring with an effective conformation to the plane of the pyrimidine ring, which could improve the activity.

Compared with the structures of APCP, BPCP and EPCP, BmPCP reserves the part of TNK-651and then was identified as a potential lead compound by the inhibition of viral infectivity in different cells. Selectivity Index reached to 84 on MT4 cell and 266 on TZM-bl cell respectively.

Here we tested the inhibitory activity of BmPCP on infection by laboratory-adapted HIV-1 strains, including HIV-1SF33 and HIV-1 1617-1 as well as HIV clinical isolates circulating in China. In China, CRF07_BC and B' have been the most commonly transmitted HIV-1 subtypes across the country[11]. Therefore, we focused on the inhibitory activity of BmPCP on Chinese epidemic HIV-1 subtype strains. The result showed that BmPCP was effective against infection by a broad spectrum of HIV-1 strains, including both R5 and X4 viruses with IC_50 _at low μM levels. BmPCP has antiviral activity on laboratory-adapted HIV strains as well as clinical isolates from Chinese AIDS patients.

Also similar to NVP, BmPCP inhibits HIV-1 infection by targeting the post entry of the HIV-1 replication cycle by time-of-additional assay. Furthermore, in vitro HIV-1 reverse transcriptase activity assays corroborated that BmPCP directly targets the process of HIV reverse transcriptase. These results suggest that BmPCP may act as a NNRTI to inhibit HIV-1 replication. BmPCP exhibited low cytotoxicity to cells that were used for testing anti-HIV-1 activity, thus having selectivity index ranging from 60 to 360, suggesting a good potential for further development of BmPCP as a new anti-HIV-1 drug.

Like NVP, BmPCP was much less effective against NNRTI drug resistant strains. However, BmPCP with a novel structure acts as a NNRTI to inhibit HIV-1 replication and can serve as a lead compound for further development of new anti-HIV-1 drugs.

## Conclusion

This study demonstrates that BmPCP has a novel structure and is effective against infection by a broad spectrum of HIV-1 strains, including both Chinese clinical isolates and laboratory-adapted HIV-1 strains with IC_50 _at low μM levels. Similar to NVP, BmPCP could significantly inhibited RT activity. BmPCP is a lead compound for the probability of future drug development.

## Methods

### Cells, viruses, and reagents

TZM-bl and MT4 cells, viruses - HIV-1 SF33, and HIV-1 1617-1, and anti-HIV-1 drugs - NVP and Enfuvirtide (T20) were all obtained from the NIH AIDS Research and Reference Reagent Program (USA). MT4 cells [12] were maintained in RPMI 1640 medium supplemented with 10% heat-inactivated fetal bovine serum (FBS), 100 U/ml penicillin and 100 μg/ml streptomycin. TZM-bl were propagated in Dulbecco's modified Eagle medium (DMEM) containing 10% (vol/vol) FBS, 100 units/ml penicillin, and 100 μg/ml streptomycin. PBMCs from two HIV-1-seronegative human donors were maintained in RPMI 1640 medium (Gibco) containing 20 U/ml of recombinant interleukin-2 (IL-2; National Institutes of Health; Bethesda, Maryland, USA), 1% penicillin and streptomycin (P/S), 2 mM glutamine and 10% FBS. The clinical viruses were isolated from patients infected by HIV-1 CRF07_BC and B' strains in China [13].

### HIV-1 inhibition assays

The inhibitory activity of BmPCP, APCP, BPCP and EPCP on infection by laboratory-adapted HIV-1 strains was tested in TZM-bl cells, MT4 cells or PBMCs, respectively, as previously described [10]. TZM-bl cells were seeded at 10^4^/well and cultured in a 96-well tissue culture plate overnight, followed by addition of 200 TCID_50 _(50% Tissue Culture Infective Dose) of HIV-1 virus. After incubation at 37°C for 2 hrs, BmPCP at graded concentrations was added. After further incubation at 37°C for 48 hrs, the luciferase activity was measured using a luciferase assay kit (Promega Corp.) according to the manufacturer's instructions. MT4 cells (4 × 10^4^/well) and PBMCs (1 × 10^5^/well) were infected by addition of 200 TCID_50 _of HIV-1 virus, followed by incubation for 2 hrs at 37°C before addition of BmPCP at serial dilutions. After further incubation at 37°C for 4 days (for MT4 cells) or 7 days (for PBMCs), HIV-1 p24 was measured using a commercial enzyme-linked immunosorbent assay (ELISA) kit (Vironostika HIV-1 Microelisa system; BioMérieux; Marcy l'Etoile, France). The inhibitory activity of BmPCP on infection by primary HIV-1 isolates was also tested in PBMCs as described above. The percent of inhibition and the IC_50 _were calculated as previously described [10].

### Cytotoxicity assay

An XTT assay, as previously described [10], was used for assessing the cytotoxicity of BmPCP to the cells tested for anti-HIV-1 activity. Briefly, BmPCP at graded concentrations was added to MT4 cells, PBMCs, and TZM-bl cells at 4 × 10^4^, 1 × 10^5 ^and 1 × 10^4 ^cells per well, respectively, followed by an incubation at 37°C for 3 days. Ten microliters of CCK-8 reagent was added to the cells. After incubation at 37°C, for 4 h to allow color development of the XTT formazan product, the absorbance of each well was then read at 450 nM in a Victor2 1420 Multilabel Counter (Wallace-PerkinElmer Life and Analytical Sciences Inc., Boston, MA). The percent of cytotoxicity and CC_50 _were calculated as previously described [10].

### Time-of-addition assay

MT4 cells were seeded at 10^4^/well and cultured in a 96-well tissue culture plate overnight, followed by addition of 200 TCID_50 _of HIV-1SF33. BmPCP (13.3 μM) and the control compounds, NVP (5 μM) and T-20 (5 μM), were respectively added 0, 0.25, 0.5, 1, 2, 4, and 8 hrs post-infection. HIV-1 p24 was measured using a commercial enzyme-linked immunosorbent assay (ELISA) kit (Vironostika HIV-1 Microelisa system; BioMérieux; Marcy l'Etoile, France).

### Assay for inhibition of HIV-1 reverse transcriptase activity

The method was described in a previous publication[14]. Briefly, Oligo(dT) 15 was immobilized via its 5'-terminal phosphate to CovaLink NH microtiter plates. The biotin-dUTP was incorporated by reverse transcriptase. The products were detected and quantified using a colorimetric streptavidin alkaline phosphatase reporter system.

## Competing interests

The authors declare that they have no competing interests.

## Authors' contributions

YH and XW performed the experiments and drafted the manuscript. LM designed the study and revised the manuscript. GY and JL designed and synthesized the compound. XY, LY, WS and TL analyzed the data. YS supervised the studies. All authors read and approved the final manuscript.
